# Linking metabolites in eight bioactive forage species to their in vitro methane reduction potential across several cultivars and harvests

**DOI:** 10.1038/s41598-022-14424-2

**Published:** 2022-06-21

**Authors:** Supriya Verma, Siegfried Wolffram, Juha-Pekka Salminen, Mario Hasler, Andreas Susenbeth, Ralf Blank, Friedhelm Taube, Christof Kluß, Carsten Stefan Malisch

**Affiliations:** 1 Institute of Plant Production and Plant Breeding, Grass and Forage Science/Organic Agriculture, Kiel University (CAU), 24118 Kiel, Germany; 2 Institute of Animal Nutrition and Physiology, Kiel University (CAU), 24118 Kiel, Germany; 3 Natural Chemistry Research Group, Department of Chemistry, University of Turku, 20014 Turku, Finland; 4 Department of Statistics, Kiel University (CAU), 24118 Kiel, Germany; 5 Grass Based Dairy Systems, Animal Production Systems Group, Wageningen University (WUR), 6700 Wageningen, The Netherlands; 6 Department of Agroecology, Aarhus University, 8830 Tjele, Denmark

**Keywords:** Chemical biology, Ecology, Plant sciences, Environmental sciences

## Abstract

An in vitro Hohenheim gas test was conducted to analyze the fermentation end-products from 17 cultivars of eight polyphenol containing forage species. The polyphenol composition and proanthocyanidin (PA) structural features of all the cultivars were analyzed with UPLC-MS/MS in leaves of vegetative or generative plants. The samples were incubated with and without polyethylene glycol (PEG, a tannin-binding agent) to separate the tannin-effect on methane (CH_4,_ ml/200 mg DM) production from that of forage quality. Sulla and big trefoil, two particularly PA rich species, were found to have the highest CH_4_ reduction potential of up to 47% when compared to the samples without PEG. However, concomitant reduction in gas production (GP, ml/200 mg DM) of up to 44% was also observed. An increase in both GP and CH_4_ production under PEG treatments, confirms the role of tannins in CH_4_ reduction. Moreover, PA structural features and concentration were found to be an important source of variation for CH_4_ production from PA containing species. Despite having low polyphenol concentrations, chicory and plantain were found to reduce CH_4_ production without reducing GP. Additionally, interspecies variability was found to be higher than intraspecies variability, and these results were consistent across growth stages, indicating the findings’ representativeness.

## Introduction

One of the main environmental issues from ruminant livestock production are enteric methane (CH_4_) emissions, which contribute to up to 40% of the total livestock sector emissions worldwide^1^**.** The radiative forcing capacity of CH_4_ is 63 times higher than that of CO_2_ over the first 20 years, and 27 times higher over the 100-year horizon. That makes CH_4_ the second most important greenhouse gas after CO_2_, regarding anthropogenic climate change^2^. On the other hand, the very shorter atmospheric lifetime of CH_4_ (around 12 years), results in a much higher potential to reduce the radiative forcing in the short term, compared to CO_2_ and N_2_O. Thus, it can provide a valuable opportunity to limit the extent of anthropogenic climate change until long term solutions are in place^3^. Although, CH_4_ emissions from ruminants cannot be completely eliminated without losing the ability of ruminants to digest cellulose, their reduction is also desirable from an economic point of view, as energy losses in the form of CH_4_ can amount up to approximately 2 to 12% of gross energy intake in ruminants^4^.

The inclusion of forages rich in plant specialized metabolites (PSMs) (e.g. tannins and saponins) in the animal diet can potentially reduce CH_4_ emissions in the ruminants^5^. Particularly tannin-rich forages (TRFs) are considered a promising solution due to their multifunctionality and their beneficial effects on animal health and productivity. Tannin-rich forages can exert beneficial and anti-nutritional effects on ruminants, however their potential depends on tannin type and concentration supplied in the feed^6^. Generally, tannins are known to bind with feed constituents such as protein, fibre, and carbohydrates. They can potentially improve protein utilization of the forages by making protein-tannin complexes in the rumen (pH = 6–7), and transporting them to abomasum (pH < 3.5) and small intestine (pH < 7) where they will eventually dissociate due to post ruminal pH shifts and release proteins. This makes the protein available for gastric or pancreatic digestion, which can reduce feed energy losses in form of CH_4_ emissions^7^. This is especially relevant for ruminant production systems in temperate regions where despite having high protein content, the conventional forages, such as lucerne (*Medicago sativa* L.) and white clover (*Trifolium repens* L.), have been associated with bloating and inefficient N utilisation^8,9^. The high extent of protein degradation in the rumen increases high urinary N excretion, which could potentially lead to increased ammonia (NH_3_) volatilization from the cattle manure and concomitantly negatively affect the environment^6^.

Tannins can be broadly classified as proanthocyanidins (PAs; *syn*. condensed tannins), hydrolysable tannins (HTs) and phlorotannins, and the latter are mainly found in marine organisms. Hydrolysable tannins can be further subdivided into gallic acid derivatives, ellagitannins and gallotannins^10^, while PAs exist in the form of polymers and oligomers of flavan-3-ol subunits, most often catechin or epicatechin together known as procyanidins (PCs), and gallocatechin and epigallocatechin (prodelphinidins, PD)^11^. Procyanidins and PDs differ from each other in terms of their hydroxylation pattern of their flavan-3-ol B-ring, with PD subunits containing three hydroxyl groups and PC subunits containing two hydroxyl groups in the B-ring^6^. The structural traits are important, particularly the percentage of PD subunits from total PAs (PD%) and the polymer size have been shown to effect the mode and extent of the desired bioactive properties from PAs^12^. Both forages rich in PAs, such as sainfoin (*Onobrychis viciifolia* L.) and birdsfoot trefoil (*Lotus corniculatus* L.), as well as forages rich in HTs, such as salad burnet (*Sanguisorba minor*), have emerged as promising species that can improve N utilisation in the ruminants while simultaneously curtailing CH_4_ emissions^13,14^. When supplied in low to moderate concentrations, tannins (both PAs and HTs) in forages can potentially improve animal performance, reduce NH_3_ volatilization from manure by redirecting N excretion from urine to feces, lower CH_4_ emissions and decrease bloating incidences in cattle^7,15^. Birdsfoot trefoil, one of the promising TRFs, has been found to increase milk yield and milk protein content compared to perennial ryegrass in Friesian cows, and improved wool growth in sheep^16^.

Several in vivo and in vitro studies have analyzed the antimethanogenic potential of TRFs; however, a high variability in the results from these studies makes it difficult to harness their benefit in the livestock systems^17^. This is likely due to the abovementioned high structural diversity of tannins, which makes it difficult to assess their bioactivity, and understand their effect on animal physiology and nutrition. It has been widely recognized that PA structural characteristics are important determinants of bioactivity, however, in the majority of animal nutrition studies the structural characteristics or the composition of PAs and HTs are rarely considered. These structural characteristics are known to vary not only across different plant species but also within their cultivars and have been found to be affected by plant’s phenological stage^18^. However, the variation within the cultivar for each species was found to be less than the variation across the species^19^. To date, no study has identified the antimethanogenic effect of several cultivars each from multiple forage species grown under identical environmental conditions and across multiple harvests to determine not only the interspecies variability but also the reproducibility of these findings within species and across harvests, which is ultimately a main prerequisite for their use in practice.

**Figure 1:**
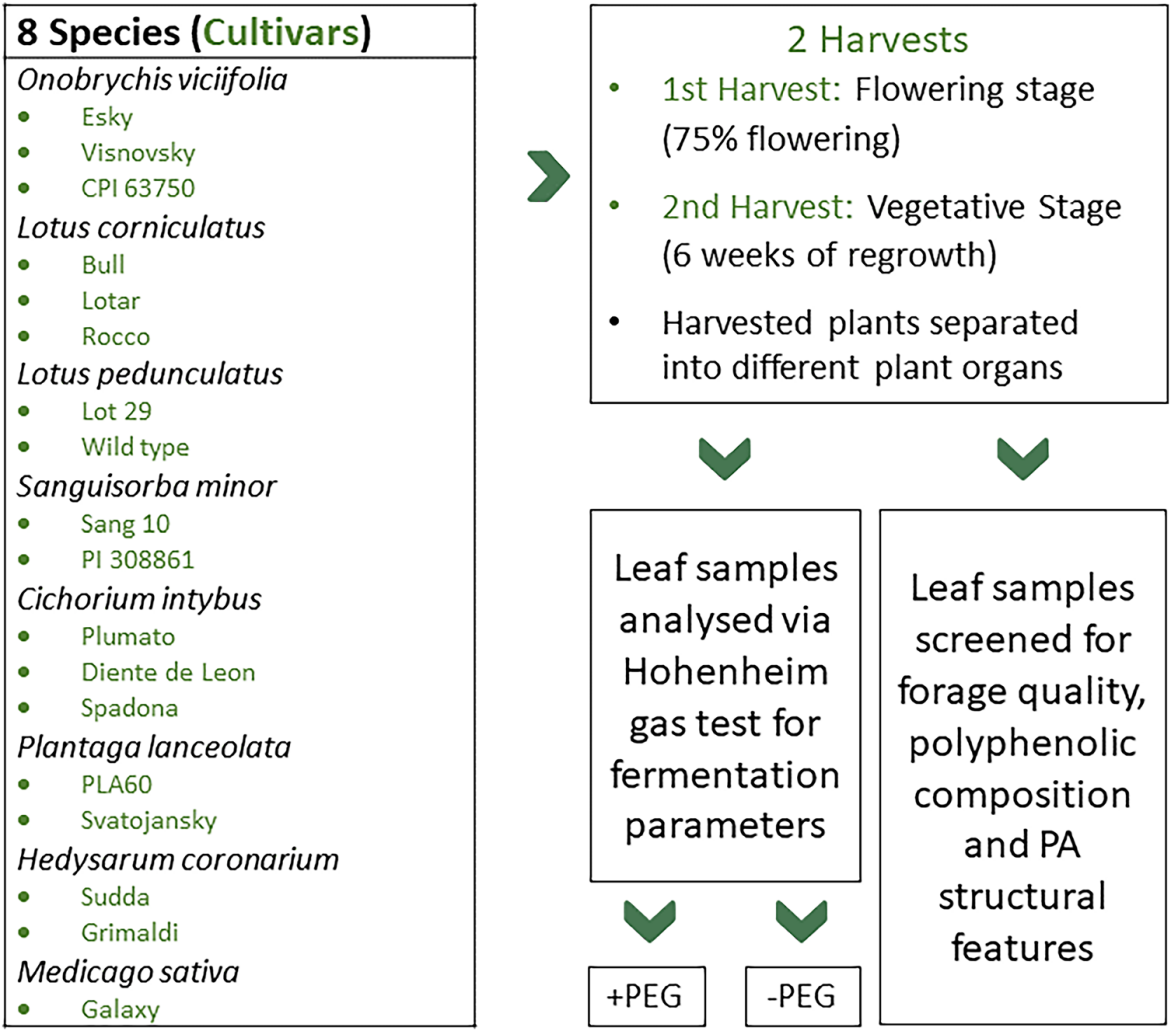
A greenhouse experiment was conducted with 17 cultivars from eight forage species. The cultivars were harvested twice at different phenological stages (Flowering and vegetative stage). Harvested *plants were* analysed for their polyphenolic composition, PA structural features, and antimethanogenic potential.

Consequently, the study aimed to assess the in vitro CH_4_ reduction potential while minimizing the aforementioned variations and accounting the often-neglected PA structural features. To our knowledge, this is the first study to analyze the CH_4_ mitigation potential of eight agronomically important temperate forage plants grown under identical environmental conditions and harvested at the same phenological stage (Fig. 1). Main objectives of this study were, (i) to analyze the inter- and intraspecies variation of the CH_4_ mitigation potential of multiple forage species, (ii) to assess the tannin-effect on the CH_4_ reduction from the TRFs, and (iii) determine the influence of PA structural features, such as PD% and PA polymer size, on the CH_4_ production.

## Results

### Forage quality and tannin composition

The forage chemical composition of the leaves varied extensively across the species. While general trends existed, and herbs had lower CP and higher NDF concentrations compared to legumes, there was a large variation across different functional groups. In general, highest CP concentration was found in big trefoil and birdsfoot trefoil cultivars (224–272 g/kg DM) when plants were harvested at the flowering stage (first harvest) (Table 1), while sainfoin cultivars had the lowest CP concentrations (157–180 g/kg DM) of all legumes and generally the highest NDF concentrations (518–548 g/kg DM). In the vegetative stage at the second harvest, however, two sainfoin cultivars (CPI 63750 and Esky) had the highest CP concentrations of all species. However, generally the differences in CP concentration decreased with the vegetative stage and no clear differences were observed between legumes and herbs at this stage. Additionally, differences between cultivars of same species were mainly observed for CP concentration for the species ribwort plantain, salad burnet and chicory in the first harvest.

**Table 1:** Chemical and polyphenol composition of the plants evaluated in the trial.

Species	Cultivar	Harvest	CP	NDF	ADF	TP	TF	TT
Chicory	Plumato	1	181	346	212	1.5^AB^	0.1^ABC^	0^A^
Chicory	Spadona	1	122	464	281	1.3^ABC^	0.1^ABC^	0^A^
Sulla	Grimaldi	1	232	496	363	22.5^D^	0^A^	22^BC^
Sulla	Sudda	1	186	495	366	25.5^D^	0.1^ABC^	25.2^B^
Birdsfoot trefoil	Bull	1	224	469	202	3.1^B^	0.7^DEF^	2.3^D^
Birdsfoot trefoil	Lotar	1	239	389	236	2.6^B^	0.5^BDE^	2.1^D^
Birdsfoot trefoil	Rocco	1	272	349	239	2.4^AB^	0.4^ABCD^	2^D^
Big trefoil	Lot 29	1	250	482	340	21.4^DE^	1.5^Fb^	19.9^BCb^
Big trefoil	Wild type	1	266	518	376	16^D^	0.9^EF^	15.1^C^
Lucerne	Galaxy	1	259	412	219	0.2^Cb^	0.2^ABCa^	0^Aa^
Sainfoin	CPI 63750	1	157	519	351	20.2^Db^	2.6^Gb^	17^BCb^
Sainfoin	Esky	1	206	530	407	18.4^Db^	2.9^Gb^	15^C^
Sainfoin	Visnovsky	1	181	549	411	20.1^D^	3.8^Gb^	15.4^C^
Ribwort plantain	PLA60	1	211	443	237	0.6^AC^	0.1^AC^	0^A^
Ribwort plantain	Svatojansky	1	129	512	319	0.3^C^	0^A^	0^A^
Salad burnet	PI 308861	1	163	523	95	43.8^E^	0.4^BCDE^	42.4^E^
Salad burnet	Sang 10	1	110	426	112	42^E^	0.3^ABCD^	40.1^E^
Chicory	Plumato	2	220	393	232	1.7^AB^	0.1^AB^	0^A^
Chicory	Spadona	2	137	449	301	1.9^AB^	0.2^AB^	0^A^
Sulla	Grimaldi	2	240	549	455	24.9^CD^	0^A^	24.5^B^
Sulla	Sudda	2	224	467	393	27.9^C^	0.1^AB^	27.7^BC^
Birdsfoot trefoil	Bull	2	229	418	247	2.3^A^	0.6^CD^	1.7^D^
Birdsfoot trefoil	Lotar	2	232	408	234	2.2^A^	0.5^CDE^	1.8^D^
Birdsfoot trefoil	Rocco	2	242	393	211	1.7^A^	0.3^BE^	1.4^D^
Big trefoil	Lot 29	2	250	554	375	12.6^DE^	0.8^Ca^	11.8^Ea^
Big trefoil	Wild type	2	229	493	344	12.4^E^	0.8^CD^	11.6^E^
Lucerne	Galaxy	2	235	433	245	0^Fa^	0^Aa^	0^Aa^
Sainfoin	CPI 63750	2	263	540	372	11.2^Ea^	1.3^Fa^	9.4^Ea^
Sainfoin	Esky	2	273	520	385	11.5^Ea^	1.7^Fa^	9.5^E^
Sainfoin	Visnovsky	2	216	554	426	13.8^E^	2.8^Ga^	10.6^E^
Ribwort plantain	PLA60	2	157	419	239	0^BFG^	0^A^	0^A^
Ribwort plantain	Svatojansky	2	122	511	342	0.2^G^	0^A^	0^A^
Salad burnet	PI 308861	2	221	552	156	49.7^H^	0.4^DE^	47.9^C^
Salad burnet	Sang 10	2	221	553	187	45.1^H^	0.3^BE^	43.3^C^

CP, crude protein (g/kg DM); NDF, neutral detergent fibre (g/kg DM); ADF, acid detergent fibre (g/kg DM); TP, total polyphenols (g/ kg DM); TF, total flavonols (sum of myricetin, quercetin and kaempferol concentration, g/ kg DM); TT, total tannins (g/ kg DM). Values identify mean concentration of the measured parameters. Within a column significant differences (*P* < 0.05) between the different cultivars for each harvest is represented by different uppercase letters and the significant differences within the harvests for each cultivar (*P* < 0.05) is represented by different lower-case letters. For CP, NDF and ADF, a pooled sample for each cultivar was used for analysis due to biomass limitation.

Significant differences among cultivars were observed for total polyphenol (TP), total flavonols (TF, sum of myricetin, quercetin and kaempferol concentration), and total tannin (TT) concentration (*P* < 0.001). The lowest TP concentration was found in chicory, ribwort plantain, and lucerne. Lucerne was found to be void of polyphenols, and was used as tannin-free negative control in the experiment. Highest TF concentrations were found in sainfoin cultivars with a range from 1.3 to 3.8 g/kg DM, while ribwort plantain cultivars generally contained no flavonols^19^. Salad burnet was the only HT containing forage in our study and had the highest TP concentrations ranging from 42 to 50 g/kg DM. Proanthocyanidin containing species were sainfoin, sulla, big trefoil and birdsfoot trefoil with concentrations ranging from 1 to 25 g/kg DM across both harvests. Proanthocyanidin structural features, PD% and mean degree of polymerisation (mDP), generally differed significantly (*P* < 0.001) across species, while differences across the cultivars of a single species were restricted to birdsfoot trefoil (Table 2)^19^. In birdsfoot trefoil, Rocco was consistently found to have significantly higher mDP values (12–14) and PD% (48–52%) than Bull (mDP: 9–10, PD%: 30–33.3%) and Lotar (mDP: 9–11, PD%: 33–39%)^19^.

**Table 2:** Proanthocyanidin (PA) concentration and the structural traits of the PA containing species evaluated in the trial.

Species	Cultivar	Harvest	PA	mDP	PD%
Sulla	Grimaldi	1	22 ± 4.9^ADE^	17.2 ± 0.9^ADE^	93.6 ± 0.5^AD^
Sulla	Sudda	1	25.2 ± 2.8^A^	19.3 ± 1.3^A^	91.4 ± 0.9^A^
Birdsfoot trefoil	Bull	1	2.3 ± 1.1^B^	9.7 ± 1B	33.3 ± 7.9^B^
Birdsfoot trefoil	Lotar	1	2.1 ± 0.5^BC^	10.6 ± 1.3^BC^	39 ± 4.1^B^
Birdsfoot trefoil	Rocco	1	2.1 ± 0.8^CD^	13.5 ± 0.8^CD^	52.3 ± 3.3^C^
Big trefoil	Lot 29	1	19.9 ± 2.4^AE^	16.7 ± 0.2^AE^	90.8 ± 1.7^AD^
Big trefoil	Wild type	1	15.1 ± 4.1^ADE^	15.8 ± 0.2^ADE^	90.5 ± 0.8^AD^
Sainfoin	CPI 63750	1	17 ± 1.2^DEb^	14.3 ± 0.7^DEb^	82.9 ± 4^D^
Sainfoin	Esky	1	15 ± 2^DE^	15 ± 0.8^DE^	81 ± 4.7^D^
Sainfoin	Visnovsky	1	15.4 ± 2.8^DE^	15.3 ± 0.5^DE^	82 ± 2.3^Db^
Sulla	Grimaldi	2	24.5 ± 4^ADE^	17.2 ± 2.3^ADE^	93.7 ± 0.4^ADE^
Sulla	Sudda	2	27.7 ± 5.5^A^	18.2 ± 2.3A	92.3 ± 1^A^
Birdsfoot trefoil	Bull	2	1.7 ± 0.7^B^	9 ± 0.9^B^	30.3 ± 1.9^B^
Birdsfoot trefoil	Lotar	2	1.8 ± 0.3^B^	9.7 ± 0.2^B^	33.8 ± 1.9^B^
Birdsfoot trefoil	Rocco	2	1.4 ± 0.7^CD^	12.8 ± 1.3^CD^	48.7 ± 4.3^C^
Big trefoil	Lot 29	2	11.8 ± 5.7^Aa^	18 ± 0.5^Aa^	89.1 ± 0.9^A^
Big trefoil	Wild type	2	11.6 ± 4.4^AE^	16.4 ± 1.2^AE^	88.3 ± 1.6^AD^
Sainfoin	CPI 63750	2	9.4 ± 3.2^BCa^	11.5 ± 1.1^BCa^	81.9 ± 4.9^CE^
Sainfoin	Esky	2	9.5 ± 2.6^BCD^	12.3 ± 2.4^BCD^	81.1 ± 2.2^CDE^
Sainfoin	Visnovsky	2	10.6 ± 2.2^CDE^	13.9 ± 0.5 ^CDE^	71.7 ± 3.8^Ea^

PA, proanthocyanidin concentration (g/kg DM); mDP, mean degree of polymerisation; PD%, prodelphinidin percentage in PA. Values identify mean concentration of the measured parameters. Within a column significant differences (*P* < 0.05) between the different cultivars for each harvest is represented by different uppercase letters and the significant differences within the harvests for each cultivar (*P* < 0.05) is represented by different lower-case letters.

### Differences in gas and methane production were observed across species and their cultivars

**Figure 2:**
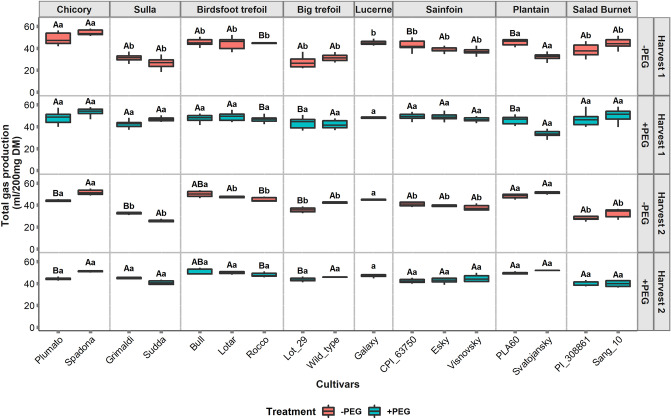
Gas production from 17 cultivars of 8 species under different treatments for two harvests. −PEG indicates the samples incubated without polyethylene glycol (PEG) and + PEG refers to the samples incubated with PEG. Different uppercase letters represent significant differences (*P* < 0.05) between the cultivars for each species, and different lowercase letters represent the significant differences within the treatments for each cultivar.

The in vitro gas production (GP) from the forages differed significantly across the cultivars (*P* < 0.001) with the GP ranging from 25 to 54 ml/200 mg DM across both harvests (Fig. 2, −PEG). At the species level, chicory had the highest GP (44 to 54 ml/200 mg DM), while sulla had the lowest GP (25 to 32 ml/200 mg DM). The variability within the cultivars of any species for GP was only observed among the cultivars of sainfoin and ribwort plantain, with the plantain cultivars showing the largest differences, as Svatojansky had produced 28% less gas compared to PLA60 in the first harvest. The effect of harvest (*P* = 0.63) on GP was not significant; however, a significant interaction effect of cultivar and harvest (*P* < 0.001) on GP was observed. This was apparent for ribwort plantain, where the cultivar Svatojansky produced 40% more gas in the second harvest compared to the first harvest and as a result, its GP then exceeded that of the cultivar PLA60. For salad burnet on the other hand, the GP was reduced in the second harvest compared to the first harvest by approx. 26%, yet this difference was homogeneous across both the cultivars.

**Figure 3:**
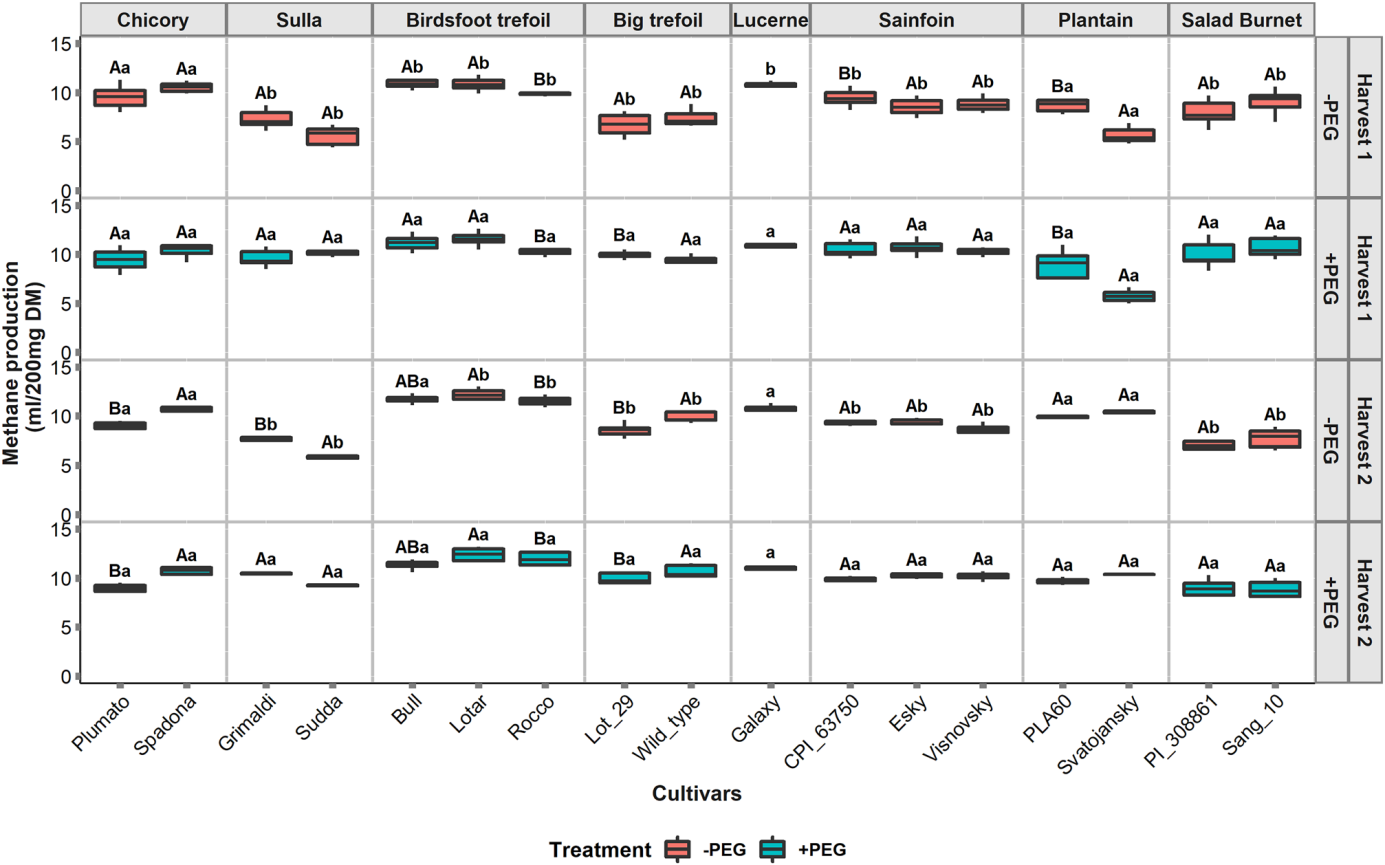
Methane production from 17 cultivars of 8 species under different treatments for two harvests. −PEG indicates the samples incubated without polyethylene glycol (PEG) and + PEG refers to the samples incubated with PEG. Different uppercase letters represent significant differences (*P* < 0.05) between the cultivars for each species, and different lowercase letters represent the significant differences within the treatments for each cultivar.

The CH_4_ production was also influenced by the plant species and their cultivars (*P* < 0.001) and the effect of harvest (*P* = 0.06) was not significant (Fig. 3, −PEG treatment). The lowest CH_4_ production was observed for the ribwort plantain cultivar Svatojansky (5.6 ml/200 mg DM) and the sulla cultivar Sudda (5.6 ml/200 mg DM), followed by the big trefoil cultivar Lot 29 (6.8 ml/200 mg DM). Despite containing PAs, CH_4_ production from birdsfoot trefoil (9–11 ml/200 mg DM) was the highest and was similar to lucerne (10.8 ml/200 mg DM). In general, the CH_4_ production from the cultivars in the first harvest was lower compared to the second harvest and there was a significant cultivar-harvest interaction (*P* < 0.001). Within species variation in CH_4_ production was observed in the cultivars from birdsfoot trefoil, sainfoin and ribwort plantain. As seen for GP, the variability in CH_4_ production between ribwort plantain cultivars was larger in the first harvest, with Svatojansky producing 35% less CH_4_ than PLA60. In the second harvest, the variation between the cultivars decreased and Svatojansky produced 5% more CH_4_ than PLA60. In birdsfoot trefoil, Rocco generally produced lowest CH_4_ and had on average 10% lower CH_4_ formation compared to other birdsfoot trefoil cultivars (Bull and Lotar). Largely, GP and CH_4_ production from the cultivars were stable across the harvests.

**Figure 4:**
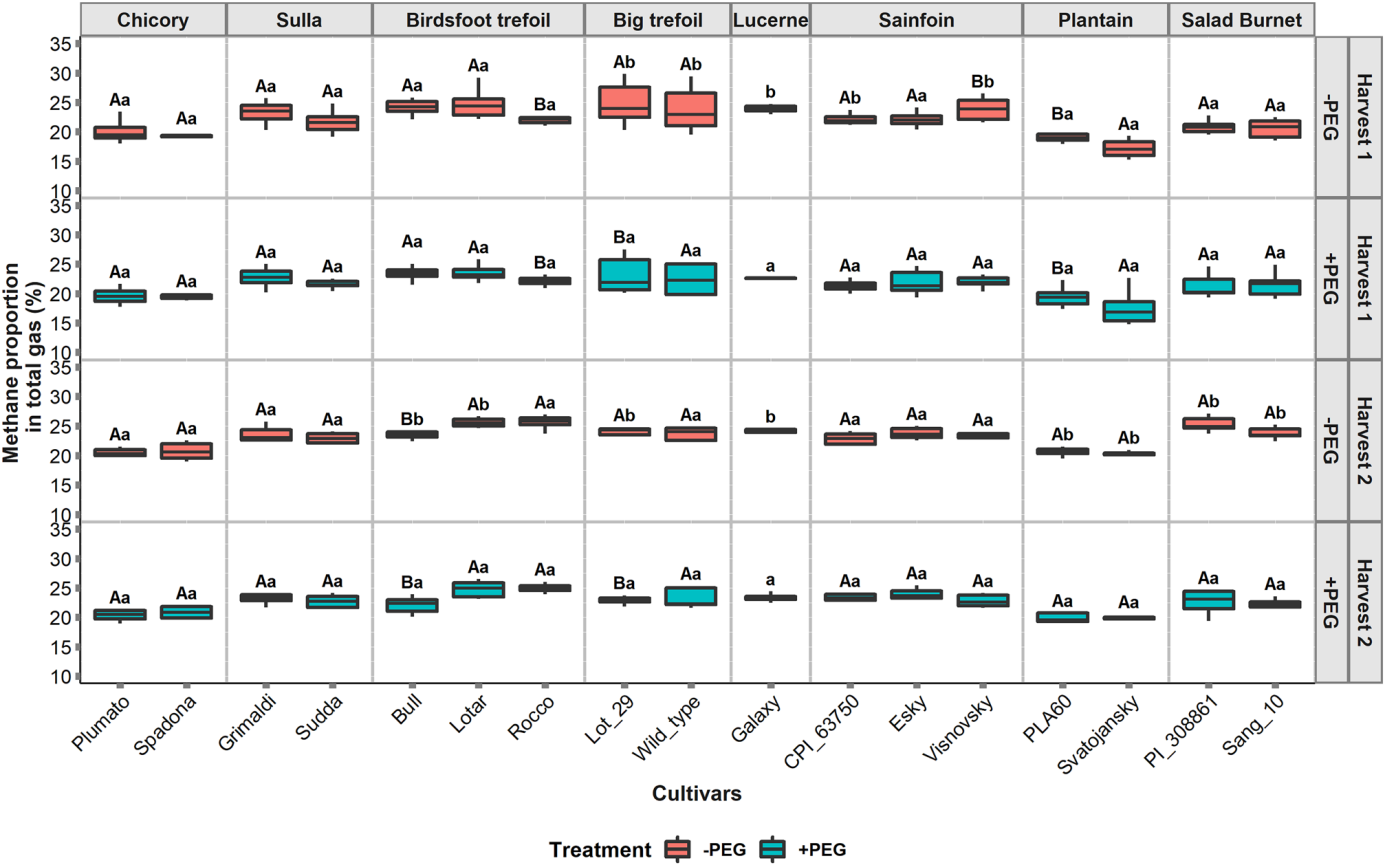
Methane percentage in total gas from 17 cultivars of 8 species under different treatments for two harvests. −PEG indicates the samples incubated without polyethylene glycol (PEG) and + PEG refers to the samples incubated with PEG. Different uppercase letters represent significant differences (*P* < 0.05) between the cultivars for each species, and different lowercase letters represent the significant differences within the treatments for each cultivar.

The cultivars were observed to differ significantly in terms of CH_4_ percentage in total gas ( MP, (*P* < 0.001)). The MP ranged from 17 to 25% in the first harvest and from 20 to 26% in the second harvest. The effect of harvest was not significant (*P* = 0.06) on MP, however the interaction between harvest and cultivar was found to be significant (*P* < 0.001). Within species variability in MP was again limited to cultivars from sainfoin, ribwort plantain, and birdsfoot trefoil (Fig. 4; −PEG treatment). Generally, the MP from tannin containing forages (20–26%) was found to be higher compared to ribwort plantain and chicory (17–21%). The lowest CH_4_ produced per unit total gas production was found in ribwort plantain followed by chicory.

In general, there was a concomitant reduction of GP and CH_4_ production from all the tannin containing species when compared to lucerne. It was observed that the decline in CH_4_ production (7–47%) with TRFs (sulla, big trefoil, salad burnet and sainfoin), was higher than the GP reduction (5–43%) when compared to lucerne (Supplementary Table S2). Contrary to that, chicory cultivars were found to increase GP (7–21%) and reduce CH_4_ production (5–25%) with respect to lucerne in both the harvests.

### Addition of PEG, significantly increased gas and methane production from tannin rich forages

The addition of PEG resulted in increments of GP (*P* < 0.001) and decrements in CH_4_ production (*P* < 0.001) during the in vitro fermentation (Figs. 2 and 3, + PEG). These changes were, however, limited to the TRFs. Ribwort plantain and chicory cultivars did not change their fermentation patterns with the addition of PEG. As a result, significant interaction effect of cultivar and PEG treatment existed for GP (*P* < 0.001) and CH_4_ production (*P* < 0.001). The GP in the + PEG treatment was found to range from 33 to 53 ml/200 mg DM, while the CH_4_ ranged from 5 to 12 ml/200 mg DM. In the first harvest, ribwort plantain cultivar Svatojansky produced the lowest CH_4_ (5 ml/200 mg DM) under the + PEG treatment, the salad burnet cultivar, Sang 10 had the lowest CH_4_ production (8.9 ml/200 mg DM) under the + PEG treatment in the second harvest. The highest change in GP and CH_4_ production caused by the PEG addition was found with sulla cultivar, Sudda, where the + PEG treatment increased GP and CH_4_ by 46 and 45 percent respectively, compared to the control (−PEG). Conversely, the lowest effect from the PEG addition in tannin containing forages, was observed on birdsfoot trefoil cultivars. While the effect of PEG treatment on the MP was significant (*P* < 0.001, Fig. 4), there was no significant interaction between treatment and harvest for the MP. There was, however, a significant effect of three-way interaction between cultivar, treatment, and harvest (*P* < 0.001) observed for MP. Still, the major variations in MP were observed for birdsfoot trefoil and sainfoin in the first harvest, while in the second harvest, a significant treatment effect was observed on the MP from birdsfoot trefoil, big trefoil, salad burnet and plantain.

### Covariates generally explain the variability of fermentation parameters

A model comparison was done by substituting fixed factors, cultivar and harvest, sequentially with measured covariates, to determine whether the measured covariates explain the formation of fermentation end products (CH_4_, GP and MP) equally well, compared to the fixed factors. Yet, replacing the fixed factors with the covariates was not able to improve the quality of the model in terms of the AIC value for neither CH_4_ (AIC = 312) nor GP (AIC = 1749; Table 3). Similarly, for MP the AIC was -646. The marginal R^2^ for CH_4_ and GP of the final models were generally high with 0.81 and 0.68, respectively. This was despite them being lower than the respective marginal R^2^ values of the reference model, with 0.89 and 0.88 for CH_4_ and GP, respectively. With regards to covariates, the covariates which significantly affected the fermentation products differed among CH_4_, GP and MP. Accordingly, the GP was significantly increased by the TF concentration, and decreased by HHDP, PD%, and mDP, while the CH_4_ production was decreased by PA concentration, CP, and mDP. The MP was significantly decreased by the PD%.

**Table 3:** The relationship between forage quality traits and the in vitro fermentation end-products (methane production (ml/200 mg DM), gas production (ml/200 mg DM), and methane percentage in total gas (%)) was estimated by stepwise regression using forward selection.

Response parameters	Covariance parameter estimate	Covariates						Model parameters	Model comparison	
		PA	CP	mDP	PD%	TF	ET		Reference Model	Covariate Based Model
Methane production	F -values	105.36	49.16	7.57				AIC	312.56	325.37
	*P* values	< 0.001	< 0.001	< 0.01				Marginal R^2^	0.89	0.81
	Coefficient	− 0.17	− 0.15	− 0.12				Conditional R^2^	0.96	0.96
Total gas production	F-values		0.05	13.32	7.26	4.82	6.61	AIC	1749.47	1781.65
	*P* values		0.83	< 0.001	< 0.01	< 0.05	< 0.05	Marginal R^2^	0.88	0.68
	Coefficient		0.18	− 5.72	− 0.55	5.17	− 177.75	Conditional R^2^	0.98	0.98
Methane percentage in total gas	F-values	0.93			5.41			AIC	− 650.08	− 646.86
	*P* values	0.34			< 0.05			Marginal R^2^	0.26	0.05
	Coefficient	0.001			− 0.001			Conditional R^2^	0.75	0.81

The table shows the model performance of the covariate- based model using measured forage quality parameters (PA, proanthocyanidins (g/kg DM); CP, crude protein (g/kg DM), mDP, mean degree of polymerisation; PD%, prodelphinidin percentage in PA; TF, total flavonols (sum of myricetin, quercetin and kaempferol concentration, gkg DM); ET, ellagitannins (g/kg DM)) as covariates and its comparison with the reference model. For the R^2^ values of the model, marginal R^2^ values (variance explained by fixed effects only) and conditional R^2^ values (variance explained by the entire model including both fixed and random terms) are presented.

## Discussion

The inclusion of forages with diverse functional groups (e.g. legumes) in mixtures generally improves the yield stability in the grassland-based livestock production systems^20,21^. Forages rich in tannins and other polyphenols provide even more benefits as they have been shown to decrease CH_4_ and N emissions from ruminant based production systems^22,23^. Although, several studies have analyzed TRFs for their effect on animal performance and CH_4_ reduction potential, the results till date have been contrasting, making it difficult to adapt the forages on the large scale. The reason for these contrasting results is assumed to be at least partially on the environmental conditions during plant growth, as these can affect tannin synthesis, as well as the results from colorimetric tannin assays that do not identify tannin structural characteristics or other compounds with potential interactions^17,24^. Simultaneously, CH_4_ emissions can vary highly across different animal species as well as the measurement methods. Both in vivo and in vitro techniques have been found to have their own set of advantages and disadvantages, leading to inconsistent results across different studies. In vivo techniques can accurately identify the impact of TRFs in an animal, yet they require large amounts of feed and are highly cost intensive thus, limiting the number of replicates or treatments. Additionally, they are still affected by the pre-diet and breed of the experimental animals hence, variability still exists across several in vivo studies^25^. On the other hand, in vitro studies are inexpensive and can be used to test several feedstuffs simultaneously with sufficient number of replicates, yet they fail to take into account the changes in rumen processes such as shifts in ruminal pH as well as ruminal buffering capacity during the fermentation of the feed^26,27^. However, in vitro studies are crucial in screening the promising feed additives on ruminal fermentation and predicting their effects in vivo. In the present study, the comparative assessment of 17 cultivars from eight agronomically promising temperate forage species, across two harvests, provides a novel insight of how the bioactive properties of these forages vary across the different species, and how stable are their antimethanogenic activity across different species and their cultivars under these harvests.

### PA structural characteristics were found to be more stable than forage chemical composition across harvests

The CP levels in legumes are known to generally be in the range of 180 to 300 g/kg of DM and this mostly exceeds animal requirements (130–170 g/kg of DM)^28^. As expected, in the present study, CP concentrations in the forages were higher in the second harvest where the plants were harvested at the vegetative stage compared to the first harvest (flowering stage). This can be explained by the much higher leaf shares in second harvest, as leaves are richer in CP than stems^29^. Higher CP content increases the digestibility of the forage, as it is easily degraded by microbes^30^. Conversely, high NDF and ADF content in the forages is known to be an indicator of lower forage digestibility^28^. A meta-analysis by Maccarana, et al.^31^ found that NDF content in the feed is negatively correlated to GP in the rumen. It can reduce the availability of rapidly fermentable carbohydrates leading to lower microbial activity in the rumen and hence a decrease in GP^32^. The flavonoid content of the cultivars was also determined, due to potential matrix effects between tannins and flavonoids, as well as because of known anti-inflammatory and antimicrobial properties that are beneficial for ruminant immunological system (Olagaray and Bradford 2019). Assessing the effect of tannins on ruminants based only on their concentration has given rise to ambiguous results in the past as structural traits of tannins have been found to play an important role in determining their bioactivity^11^. Consequently, PA structural features were also analyzed in addition to concentration as they have been hypothesised to play an important role in determining their bioactivity, yet are rarely analysed in the animal studies. In the present study, these features were found to be stable across the harvests, and within the cultivars of the same species, the differences were comparatively lower than the variability across the species.

### Methane and gas production from TRFs were consistently lower than lucerne in both the harvests

Numerous studies have demonstrated that TRFs reduce enteric fermentation in ruminants, resulting in lower GP and CH_4_ production^33–35^. In the first harvest, across all the species with varying forage quality and plant metabolite composition, the largest reductions in CH_4_ and GP were observed from TRFs, which were sulla, big trefoil, sainfoin and salad burnet, in comparison with lucerne. This could be attributed to the high TT concentration in these forages compared to lucerne. This is in line with the previous studies, where TRFs reduced the fermentation in the ruminants resulting in lower GP^34^. Tannins have the propensity to bind with protein and carbohydrates (both structural and non-structural carbohydrate), preventing fermentation and degradation of the molecules, thus lowering GP^35^. A study by Jayanegara et al.^36^ found that HTs are more potent compared to PAs. In the present study, however, this was not confirmed, as both the GP and CH_4_ production of PI 308861 (salad burnet) were found to be higher than those of Sudda (sulla) and Lot 29 (big trefoil). This was despite the higher TT and TP concentrations in salad burnet, which reaffirms the results from studies, indicating that the extent of CH_4_ reduction from TRFs does not only depend on tannin concentration but also on the tannin source and structural features^37,38^. At the animal scale, the differences between PAs and HTs bioactivity is also likely to be dependent on their fate in biological systems. Proanthocyanidins exist in the form of large polymers, enabling them to bind to the feed components strongly and they do not get easily degraded in the rumen. On the other hand, HTs have been shown to undergo hydrolysis, hence degrading to form low molecular weight phenolics in the rumen, and can thereby, lose part of their bioactivity^39,40^. However, the enzymatic or auto-oxidation of the initial HTs or their hydrolysis products could also produce large bio-oligomers or polymers with tannin-like functions in the rumen or small intestine^39^. Consequently, this makes it difficult to assess the fate of tannins in biological systems from in vitro studies alone. A general trend of higher GP and CH_4_ production, when plants were harvested at the vegetative stage (second harvest), was recorded compared to when they were harvested at the flowering stage (first harvest) which could be a result of lower tannin content in the plants in the second harvest. Furthermore, lower content of instantly fermentable fractions, such as water soluble carbohydrates in the leaves at the flowering stage can result into lower GP^41^. Interestingly, the species with lower TP and TT content, such as ribwort plantain and chicory, were also found to reduce CH_4_, but it was not accompanied by reduction in GP. This could result from PSMs other than tannins, found in these forages. A study by Navarrete, et al.^42^, found that chicory is rich in inulin and sesquiterpene lactones and, ribwort plantain leaves are known to contain acteoside, aucubin, and catapol.

Additionally, it is important to look at the CH_4_ percentage (MP) in the total gas, as this can be used as an indicator of the potential CH_4_ emissions per unit of organic matter (OM) degraded. Due to the concomitant reductions in GP and CH_4_ from TRFs, their MP ranged between 21 and 26% and hence was comparable to that of lucerne (24%) (Supplementary, Table S3). Chicory and ribwort plantain (17–21%) had the lowest MP compared to the other tested species in this study. The reduction in the CH_4_ from these species was not accompanied by the reduction in GP indicating their higher potential for CH_4_ abatement in ruminants without negatively affecting ruminal fermentation.

### PEG treatment affirmed the antimethanogenic activity of TRFs, however no influence on methane percentage in total gas was observed

The inclusion of PEG as a tannin inactivating agent, enabled the quantification of the specific tannin-effect from different species on the ruminal fermentation in vitro. Polyethylene glycol can inactivate tannins by displacing proteins from tannin-protein complexes, and binding with tannins to form tannin-PEG complexes. Increased GP and CH_4_ production from TRFs under + PEG treatment could be a result of high availability of protein after being dissociated from tannin-protein complexes. The increased availability of N compounds that can no longer bind to tannins with the addition of PEG leads to more substrate for microbial degradation and higher microbial growth. This in turn results into increased ruminal fermentation and higher GP^38,43^. Simultaneously, as the tannins are inactive they can no longer influence the methanogens in the rumen which could affect CH_4_ production from these species. As expected, no differences in GP and CH_4_ production were observed from the samples of ribwort plantain, lucerne and chicory, due to the absence of tannins in these species. Generally, the variation in the extent of GP and CH_4_ reduction from across the different TRFs under −PEG and + PEG treatments was in line with the variation in tannin concentration and composition. Accordingly, stronger PEG-treatment effect was observed in cultivars with higher tannin concentrations, which is in line with the studies by Basha et al.^43^ and Jayanegara et al.^35^.

Although, it is evident that tannins reduce CH_4_ emissions, the mode of action by which they reduce CH_4_ in ruminants is still not well understood. A study by Hassanat and Benchaar^33^ found that the PA (acacia and quebracho) and HT (chestnut and valonea) extracts had a more pronounced effect on rumen methanogenesis compared to the substrate degradation^33^. Tannins are found to modify the rumen microflora by either directly inhibiting methanogenic population or indirectly affecting methanogen-protozoa symbiosis by inhibiting protozoal population in rumen^7,17^. In a study by O'Donovan and Brooker^44^, PAs from acacia were able to change the morphology of *Streptococcus bovis*, *Butyrivibrio fibrisolvens* and *Prevotella ruminicola*, and, hence, affected their activity. Additionally, HTs and PAs from different plant sources were also found to decrease CH_4_ production by suppressing the methanogenic archaea and protozoal populations in vitro^45^.

### PA structural features were able to explain the variability in methane production from PA containing species but model was not improved

As mentioned above, not only the differences between HT and PA are relevant, but also their structural characteristics affect their potential bioactivity. Both mDP and PD% are among the most relevant features of PAs, determining their protein precipitation activity, which in turn is linked to anthelmintic and antimethanogenic bioactivity of PAs^11,46^. Hence, in the present study, a stepwise regression was performed to analyze which forage parameters were best suited to explain the total gas and CH_4_ production. By identifying covariates as potential predictors, fermentation patterns and CH_4_ reduction potential could be estimated from plant traits. This would enable extrapolations to any other species in terms of their bioactivity and would potentially help in predicting their influence on ruminants. However, the updated model (covariate-based model) with forage composition parameters as covariates did not improve the base model in terms of AIC value. Nevertheless, the conditional R^2^ for CH_4_ and GP remained the same and even increased for MP. This indicates that the overall updated model, including both fixed and random effects, was able to explain the variance of CH_4_ and GP in the present study for PA containing species. However, in order to predict the fermentation parameters, cultivar remains the better estimate compared to the sum of all covariables, indicating that due to the large number of factors with interconnected effects, the tannin source remains more relevant compared to the traits alone. This could also arise from the fact that in addition to PAs, plants may produce other bioactive PSMs such as terpenes, organic acids, saponins and others which can also influence the CH_4_ production from the substrate^6,47^ which were not quantified in this study. Furthermore, the influence of matrix effects between tannins and other PSMs on tannin bioactivity remains unexplored. Still, the model quality was sufficient to identify parameters which had a strong influence on the in vitro fermentation end-products. Particularly in case of CH_4_, the variables CP, PA concentration and mDP, were found to exhibit a negative relationship with CH_4_ production. This is in accordance with the study by Hatew et al.^48^, which observed that the polymer size (mDP) was an important determinant of in vitro CH_4_ and GP production from the substrate. With regards to MP, however, both PA and PD% were found to have limited influence as indicated by their near-zero coefficient and low marginal R^2^ (0.05). One solution to improve the predictive capabilities of CH_4_ reductions from tannin traits compared to forage quality traits might be the inclusion of tannin extracts in in vitro incubation instead of the whole plant sample.

### The tested forage species are promising alternatives as sustainable feedstock in livestock production systems

The results in the present study underpin that TRFs have high potential for their use in livestock production as their CP content across both harvests was comparable to lucerne. This was further demonstrated by the GP production in the presence of PEG, which did not vary significantly from lucerne. The inclusion of PEG treatment further established the role of tannins in the antimethanogenic activity of TRFs. Hydrolysable tannins have been observed to affect methanogens in the rumen, however, the mode of action of PA containing forages has been found to be variable^24^. A significant treatment effect was observed when sulla and big trefoil were employed as a substrate, indicating their higher antimethanogenic activity which resulted from their high PA content and potent PA structural characteristics. Sulla is a highly palatable forage and its supplementation can reportedly reduce the dependency of the livestock production systems on proprietary anthelmintics^49^. The supplementation of fresh fed big trefoil was found to reduce CH_4_ emissions by 26% (g CH_4_ kg^−1^ DM intake) compared to lucerne in sheep^50^. However, the nutritional value of sulla and big trefoil need to be evaluated before providing them as a substantial feed component. Reportedly, owing to the high tannin content, sulla and big trefoil can compromise both, N digestibility and microbial activity in rumen which leads to lower GP, in addition to CH_4_ reduction^51^. Furthermore, forages containing PAs with high mDP and high PD% (big trefoil, sulla and sainfoin) are able to reduce protein degradation more effectively than PC-rich forages such as birdsfoot trefoil^38^. Additionally, PA concentration in the leaves of birdsfoot trefoil in our study, was found to be too low (approx. 0.1%) to exhibit detectable antimethanogenic activity. The concomitant reduction in GP and CH_4_ from TRFs in the present study, makes it essential to research on how these forages can be incorporated in animal feed and in which proportion, to exploit their beneficial effects. A study by Orlandi, et al.^52^ found that even though total-tract N digestibility by PA inclusion decreased linearly with the increased concentration, there was a linear increase in N retention and the efficiency of N utilization was improved with the inclusion of tannin extracts of *Acacia mearnsii* until the concentration of 18 g/kg DM, implying that the positive impact of duodenal flux of amino acid supply can outweigh the reduction in protein digestibility when tannins are supplied at optimum levels^52^. Although more research is required to determine the relationship between PAs and post ruminal amino acid availability, it is well established that low to moderate PA concentrations leads to increased rumen undegraded protein and post ruminal amino acid flux which improves the N utilization efficiency in ruminants^6^.

Furthermore, the mechanism by which tannins exert their antimethanogenic activity needs to be investigated. There is a possibility that rumen microbiome can get adapted to these bioactive compounds which could influence their effect in the long term and, thus, their CH_4_ reduction potential^6,53^. However, the research on this topic is still limited, and further research is warranted to understand long-term feeding effects of tannin-containing legumes rumen microbiome and their antimethanogenic activity. The stability of PA structural features across the different harvests is a promising ground to include PA structural features for future structure–activity relationship studies of tannins.

Additionally, this study also showed that underutilized forbs, such as chicory and ribwort plantain, have a high CH_4_ reduction potential despite being void of tannins. The CH_4_ reduction from these species did not result in a negative impact on GP when compared to the other tested species in this study. This is in line with an in vivo study by Niderkorn et al.^54^, which found that sheep produced 23% lower CH_4_ per kg DM intake with pure chicory compared to when fed with pure perennial ryegrass (*Lolium perenne* L., cv. AberAvon) which no significant differences in the DM digestibility. The bioactive compounds, aucubin and acteoside, in these forages are found to reduce net NH_3_ production during ruminal fermentation, and increase potential total GP^42^. Both chicory and ribwort plantain are promising alternatives to produce high quality feed to overcome feed-deficits during dry seasons due to their drought tolerance, and their inclusion in grass-clover mixtures can enhance above- and below-ground primary production in temporary grasslands^55,56^. Furthermore, they are known to benefit animal health due to their antimicrobial and anthelmintic properties which could be attributed to the presence of the PSMs other than polyphenols which were not analyzed in this study^23,57^.

## Conclusions

In summary, low intraspecies variation compared to interspecies variation in antimethanogenic potential of these species is a promising basis for breeding and selection opportunities. Our study found that the intraspecies variability was much lower compared to the interspecies variability in terms of polyphenolic composition as well as PA structural features, and these features were generally stable across the harvests. Furthermore, these variations were translated to the antimethanogenic activity of the species. The reproducibility of the bioactive effects of TRFs across both the harvests provides a promising outlook of their potential to reduce CH_4_ in the livestock production systems. However, as the measurements were conducted under controlled conditions, it is crucial to assess their potential under field conditions. Additionally, inclusion level of these species in ruminant diet should also be assessed to prevent their negative effects on digestibility and animal performance. Inclusion of PEG treatment was able to establish the role of tannins in the antimethanogenic activity of TRFs. Furthermore, it was illustrated that the antimethanogenic property of the PA containing forages is at least in part a consequence of PA structural characteristics, as the highest CH_4_ reduction was observed from sulla containing highest PD% and mDP value. Moreover, the stability of PA structural characteristics across the harvests and less variation within the cultivars of the same species than across the species, provides an opportunity for breeding and selection programmes based on PA structural characteristics. Hence, follow up studies to screen the species with high antimethanogenic property for their variation in PA structural features and bioactivity are suggested, which can in turn improve the predictability of structure–activity relationship of tannins. Assessing the effect of PA structural features in addition to PA concentration on the bioactivity of these forages could be a useful criterion to understand the diverse effects of different plant sources containing similar PA concentration, as these differences have been shown to produce quantifiable effects.

## Methods

### Experimental design

A greenhouse experiment was conducted with 17 different forage cultivars from 8 species (Table 4). The seeds for the plants were obtained from the seedbank of IPK Leibniz Plant Genetics and Crop Plant Research, Gatersleben. The plants were harvested twice at different developmental stages. In the first harvest, plants were harvested at the flowering stage and in the second harvest, which was done after a 6-week regrowth interval, plants were harvested at the vegetative stage. The plants were grown in flower pots of 24 × 24 × 40 cm^3^ (LxWxH) each and 5 plants were sown in each pot. Each cultivar had 4 pots as replicates. The pots were arranged in a complete randomized design and reflective foils were installed to ensure even lighting. For details see Verma et al.^19^. The plants were consequently separated into leaves, stems and flowers. The samples were freeze dried and stored at − 80 °C for further analysis. The leaf samples were subsequently ball milled, and stored at − 80 °C to preserve the structural integrity of the polyphenols in the sample. The average monthly temperature in the greenhouse during the plant growth ranged from 24 to 27 °C (March to August). The experimental research on plants complies with the relevant institutional, national, and international guidelines and legislation.

**Table 4:** The plant species and their cultivars used in the greenhouse experiment.

Species	Cultivar	Common name	Plant family
*Onobrychis viciifolia*	Visnovsky	Sainfoin	Fabaceae
*Onobrychis viciifolia*	CPI 63750	Sainfoin	Fabaceae
*Onobrychis viciifolia*	Esky	Sainfoin	Fabaceae
*Lotus corniculatus*	Bull	Birdsfoot trefoil	Fabaceae
*Lotus corniculatus*	Lotar	Birdsfoot trefoil	Fabaceae
*Lotus corniculatus*	Rocco	Birdsfoot trefoil	Fabaceae
*Lotus pedunculatus*	Lot 29	Big trefoil	Fabaceae
*Lotus pedunculatus*	Wild type	Big trefoil	Fabaceae
*Sanguisorba minor*	Sang 10	Salad burnet	Rosaceae
*Sanguisorba minor*	PI 308861	Salad burnet	Rosaceae
*Cichorium intybus*	Plumato	Chicory	Asteraceae
*Cichorium intybus*	Spadona	Chicory	Asteraceae
*Plantaga lanceolata*	PLA60	Ribwort plantain	Plantaginaceae
*Plantaga lanceolata*	Svatojansky	Ribwort plantain	Plantaginaceae
*Hedysarum coronarium*	Sudda	Sulla	Fabaceae
*Hedysarum coronarium*	Grimaldi	Sulla	Fabaceae
*Medicago sativa*	Galaxy	Lucerne	Fabaceae

### Chemical composition of forages

Nitrogen concentration of the forages was determined by the DUMAS rapid combustion method using a CN elemental analyser (Vario Max Elementar Analysensysteme, Hanau, Germany). Crude protein (CP) was calculated by multiplying the N concentration with 6.25. The concentrations of neutral detergent fibre (NDF) was analysed according to Van Soest detergent procedure^58^ and AOAC method 973.18. The methods were adapted to be used with fibre analyser Ankom A220 (Ankom Technology, Macedon, NY, USA).

### Polyphenol and tannin analysis with UPLC-DAD-MS/MS

The leaf tannin extracts were prepared for the UPLC-DAD-MS/MS as described by Verma, et al.^19^. The samples were analyzed with an Acquity UPLC (Waters Corp., Milford, MA, USA) coupled with a XEVO triple-quadrupole mass spectrometer (Waters Corp., Milford, MA, USA) as described by Engström et al.^59^. The system consisted of a sample manager, a binary solvent manager and a column, coupled with diode-array detector. The UV spectra was recorded. For the elution, acetonitrile (A) and 0.1% aqueous formic acid (B) were used with a constant flow rate of 0.5 mL min^–1^ with the following elution profile: 0–0.5 min, 0.1% A (isocratic); 0.5–5.0 min, 0.1–30% A (linear gradient); 5.0–8.0 min, 30–45% A (linear gradient); 8.0–11.5 min, column wash and stabilization. The data was recorded from 0 to 6 min for UV–Vis (190–500 nm) and MS data (m/z 100 to 2000). The specifications for the negative electrospray ionization are as follows; capillary voltage: 2.4 kV, desolvation temperature: 650 °C, source temperature: 150 °C, flow rate of desolvation and cone gas (N_2_): 1000 and 100 L/h, respectively, and collision gas: argon.

### Hohenheim gas test

From the 4 pots per cultivar, due to analytical constraints, 3 pots were selected for analysis with the Hohenheim gas test (HGT) in the first harvest. For the same reason, in the second harvest, the biomass from all pots for each cultivar was pooled. The GP and CH_4_ production from the samples were analyzed using  in vitro HGT^60^. The plant samples (200 ± 1 mg) were added to 100 mL calibrated glass syringes (Haeberle Labortechnik, Lonsee-Ettlenschieß, Germany) and rotated inside the incubator (39 °C) for 24 h. The samples were run in triplicates and each run was performed on two different days leading to 6 replicates per sample. Additionally, to standardize each run, four blanks (no plant material) as well as hay and concentrate standards from the Institute of Animal Science, University of Hohenheim, were used as a reference, and were included in triplicates. The animals were fed a ration consisting of grass hay (3 kg) and concentrate (3 kg) divided into 2 meals (7 a.m. and 4 p.m.). Ruminal fluid was collected prior to morning feeding from two ruminally-cannulated, non-lactating crossbred heifers (Jersey × German Black Pied). It was filtered with cheese cloth into a prewarmed insulated flask, immediately transferred to the lab, and mixed with a freshly prepared buffer solution at 1:2 ratio (v/v). The mixture was continuously stirred and flushed with CO_2_ while maintained in a water bath (39 °C). The buffered ruminal fluid (30 ml) was added to the syringes containing plant samples and syringes were placed back in the incubator with the rotor set to about one rotation per minute during the period of incubation (24 h). The GP and CH_4_ production from the samples were measured at the time interval of 8 and 24 h. The GP was recorded from the scale printed onto the syringes and methane concentration in the fermentation gas was measured with an infrared spectrometer (Methan AGM 10, Firma Sensors Europe, Ratingen, Germany). The infrared spectrometer was calibrated with pure nitrogen gas (zero point) and a gas mixture of methane and CO_2_ (60:40) as standard. The measured GP volume from the samples after 24 h was corrected with an average of the factors obtained from hay and concentrate standards. These constant values were calculated based on the measured and the targeted GP values of 45.9 ml/200 mg DM and 65.13 ml/200 mg DM after 24 h incubation of hay and concentrate standards, respectively.

Additionally, to evaluate the specificity of tannin effect on the gas production parameters, PEG treatment was included to attenuate the effect of tannin on fermentation. The samples were incubated with 200 mg PEG (MW: 6,000 kDa). The PEG treatments were referred as + PEG (samples with PEG) and −PEG (samples without PEG).

### Statistical analysis

The statistical analysis was done with using the software R^61^. A linear mixed model was used with cultivars, harvests, treatment, and their interactions (two- and threefold) as fixed factors. Analytical replicates from the HGT per experimental pots and per date of experiment were used as random factor. Graphical residual analysis was performed and based on that, the data was assumed to be normally distributed and heteroscedastic. An ANOVA and multiple contrast tests were conducted to compare the significance of the influence factors on different levels. All tests of significance were made at *P* < 0.05.

Second, to assess whether the measured forage quality parameters of PA containing species can explain the fermentation end products, a (forward) model selection was done based on the AIC value (Akaike information criterion) as described by Venables and Ripley^62^. Therefore, the same model as above was used here as a reference model containing a subset of only PA containing species and the “−PEG” values (without the corresponding PEG treatment effects). In the reference model, the fixed factors were cultivar (only PA containing species), harvest and their interaction effect, and the random effect were the analytical replicates from the HGT per experimental pots and per date of experiment. Subsequently, the previous fixed factors were removed and replaced consecutively with forage trait derived covariates (forage quality and tannin traits). The decision for or against a certain covariate was also based on its significance and its variance inflation factor (VIF; < 5) until the optimal model with the lowest complexity was achieved. The final, covariate-based model was then compared with the reference model.

## Supplementary Information

Supplementary Tables.

## Data Availability

Data sharing not applicable. The data generated during the study is included within this article and supplementary information.
