# Supplementary material for: Linking metabolites in eight bioactive forage species to their in vitro methane reduction potential across several cultivars and harvests
Source: Sci Rep. 2022 Jun 21;12:10454. doi: 10.1038/s41598-022-14424-2 (PMC9213545; doi:10.1038/s41598-022-14424-2)
Supplement: Supplementary file 1 — Supplementary Tables. [file 41598_2022_14424_MOESM1_ESM.docx]

**Linking metabolites in eight bioactive forage species to their *in vitro* methane reduction potential across several cultivars and harvests**

Supriya Verma ^1,*^, Siegfried Wolffram ^2^, Juha-Pekka Salminen ^3^, Mario Hasler^4^, Andreas Susenbeth ^2^, Ralf Blank ^2^ Friedhelm Taube ^1,5^, , Christof Kluß ^1^ and Carsten Stefan Malisch ^1,6^

^1^Institute of Plant Production and Plant Breeding, Grass and Forage Science/Organic Agriculture, Kiel University (CAU), 24118 Kiel, Germany

^2^Institute of Animal Nutrition and Physiology, Kiel University (CAU), 24118 Kiel, Germany;

^3^ Natural Chemistry Research Group, Department of Chemistry, University of Turku, Vatselankatu 2, FI-20014 Turku, Finland

^4^Department of Statistics, Kiel University (CAU), 24118 Kiel, Germany

^5^Grass Based Dairy Systems, Animal Production Systems Group, Wageningen University (WUR), 6705 Wageningen, The Netherlands

^6^Department of Agroecology, Danish Institute of Agricultural Sciences, P.O. Box 50, DK-8830 Tjele, Denmark

^*^ Corresponding author: Email address: sverma@gfo.uni-kiel.de, Tel: +49 431 880 2137

**Supplementary Table S1.** Gas production from different species and cultivars across the two harvests with and without polyethylene glycol (PEG, a tannin binding agent) treatments and the reduction potential of the tested cultivars with respect to control. Red_MS refers to the reduction in gas production from the cultivar when compared with lucerne, Red_PEG refers to reduction in gas production from the cultivar in the absence of PEG (-PEG) compared to the treatment with the addition of PEG (+PEG). Numbers between brackets correspond to the standard error of the mean (SEM).

| **Species** | | **Cultivar** | **Harvest 1** | | | | | | | | | **Harvest 2** | | | | | |
| --- | --- | --- | --- | --- | --- | --- | --- | --- | --- | --- | --- | --- | --- | --- | --- | --- | --- |
|  |  |  | **-PEG**  **(ml/200mg DM)** | | **+PEG**  **(ml/200mg DM)** | | **Red_MS (%)** | | **Red_PEG (%)** | | **-PEG**  **(ml/200mg DM)** | | **+PEG**  **(ml/200mg DM)** | | **Red_MS (%)** | | **Red_PEG (%)** |
| Chicory | | Plumato | 47.93 (1.23) | | 48.12 (1.16) | | -6 | | 0 | | 44.07 (0.35) | | 44.30 (0.77) | | 2 | | 1 |
| Chicory | | Spadona | 54.42 (0.56) | | 53.40 (0.78) | | -20 | | -2 | | 51.65 (1.06) | | 51.35 (0.55) | | -15 | | -1 |
| Sulla | | Grimaldi | 31.27 (0.77) | | 42.11 (0.80) | | 31 | | 26 | | 32.48 (0.52) | | 45.20 (0.81) | | 28 | | 28 |
| Sulla | | Sudda | 26.26 (1.06) | | 47.02 (0.46) | | 42 | | 44 | | 25.62 (0.46) | | 40.78 (0.87) | | 43 | | 37 |
| Birdsfoot trefoil | | Bull | 45.25 (0.72) | | 47.67 (0.68) | | 0 | | 5 | | 50.10 (1.21) | | 51.54 (1.20) | | -11 | | 3 |
| Birdsfoot trefoil | | Lotar | 44.58 (1.32) | | 49.26 (0.76) | | -1 | | 8 | | 47.73 (0.49) | | 49.97 (0.55) | | -6 | | 4 |
| Birdsfoot trefoil | | Rocco | 44.75 (0.24) | | 47.04 (0.78) | | 4 | | 5 | | 45.05 (0.84) | | 47.93 (0.84) | | 0 | | 6 |
| Big trefoil | | Lot 29 | 27.54 (1.36) | | 43.23 (1.05) | | 39 | | 36 | | 35.60 (1.01) | | 43.60 (0.76) | | 21 | | 18 |
| Big trefoil | | Wild type | 31.27 (0.83) | | 42.21 (1.13) | | 31 | | 25 | | 42.38 (0.36) | | 46.08 (0.56) | | 6 | | 8 |
| Lucerne | | Galaxy | 45.16 (1.36) | | 48.1 (0.3) | | 0 | | 6 | | 44.78 (0.41) | | 47.35 (1.23) | | 1 | | 5 |
| Sainfoin | | CPI 63750 | 42.81 (0.95) | | 49.29 (0.72) | | 5 | | 13 | | 40.83 (0.91) | | 42.33 (0.83) | | 9 | | 4 |
| Sainfoin | | Esky | 38.74 (0.77) | | 49.29 (0.90) | | 14 | | 20 | | 39.56 (1.01) | | 42.77 (1.03) | | 12 | | 7 |
| Sainfoin | | Visnovsky | 36.94 (0.76) | | 46.47 (0.44) | | 18 | | 21 | | 37.63 (1.11) | | 44.75 (1.43) | | 16 | | 16 |
| Plantain | | PLA60 | 45.44 (0.63) | | 46.12 (0.82) | | -1 | | 1 | | 48.07 (0.86) | | 48.70 (1.03) | | -7 | | -4 |
| Plantain | | Svatojansky | 32.46 (0.59) | | 33.33 (0.77) | | 28 | | 3 | | 51.55 (0.66) | | 51.98 (0.40) | | -14 | | 1 |
| Salad burnet | | PI 308861 | 38.48 (1.24) | | 46.90 (1.30) | | 15 | | 18 | | 27.97 (0.86) | | 39.65 (1.00) | | 38 | | 29 |
| Salad burnet | | Sang 10 | 44.28 (1.10) | | 49.98 (1.13) | | 2 | | 11 | | 32.60 (1.75) | | 39.75 (1.27) | | 28 | | 18 |
|  | **Factors** | | | **F-value** | | ***p*-value** | |  |  | **Factors** | | | | **F-value** | | ***p*-value** | |
|  | Cultivar | | | 334.35 | | <0.001 | |  |  | Cultivar x Harvest | | | | 87.12 | | <0.001 | |
|  | Treatment | | | 611.19 | | <0.001 | |  |  | Treatment x Harvest | | | | 22.39 | | <0.001 | |
|  | Harvest | | | 0.02 | | 0.89 | |  |  | Cultivar x Treatment x Harvest | | | | 5.78 | | <0.001 | |
|  | Cultivar x Treatment | | | 97.52 | | <0.001 | |  |  |  | | | | |  | |  |

**Supplementary Table S2**. Methane production from different species and cultivars across the two harvests with and without polyethylene glycol (PEG, a tannin binding agent) treatments and the reduction potential of the tested cultivars with respect to control. Red_MS refers to the reduction in methane production from the cultivar when compared with lucerne, Red_PEG refers to reduction in methane production from the cultivar in the absence of PEG (-PEG) compared to the treatment with the addition of PEG (+PEG). Numbers between brackets correspond to the standard error of the mean (SEM).

| **Species** | | **Cultivar** | **Harvest 1** | | | | | | | | | **Harvest 2** | | | | |
| --- | --- | --- | --- | --- | --- | --- | --- | --- | --- | --- | --- | --- | --- | --- | --- | --- |
|  |  |  | **-PEG**  **(ml/200mg DM)** | | **+PEG**  **(ml/200mg DM)** | | **Red_MS (%)** | | **Red_PEG (%)** | | **-PEG**  **(ml/200mg DM)** | | **+PEG**  **(ml/200mg DM)** | | **Red_MS (%)** | **Red_PEG (%)** |
| Chicory | | Plumato | 9.51 (0.25) | | 9.44 (0.22) | | 12 | | -1 | | 9.05 (0.15) | | 8.97 (0.23) | | 16 | -1 |
| Chicory | | Spadona | 10.51 (0.10) | | 10.41 (0.13) | | 3 | | -1 | | 10.67 (0.11) | | 10.72 (0.17) | | 1 | 0 |
| Sulla | | Grimaldi | 7.28 (0.18) | | 9.56 (0.16) | | 33 | | 24 | | 7.66 (0.08) | | 10.47 (0.07) | | 29 | 27 |
| Sulla | | Sudda | 5.62 (0.19) | | 10.13 (0.06) | | 48 | | 44 | | 5.85 (0.07) | | 9.25 (0.04) | | 46 | 37 |
| Birdsfoot trefoil | | Bull | 10.91 (0.09) | | 11.17 (0.14) | | -1 | | 2 | | 11.75 (0.17) | | 11.34 (0.22) | | -9 | -4 |
| Birdsfoot trefoil | | Lotar | 10.87 (0.15) | | 11.53 (0.13) | | -2 | | 4 | | 12.20 (0.24) | | 12.40 (0.29) | | -13 | 2 |
| Birdsfoot trefoil | | Rocco | 9.85 (0.05) | | 10.28 (0.07) | | 9 | | 6 | | 11.52 (0.19) | | 11.97 (0.30) | | -6 | 4 |
| Big trefoil | | Lot 29 | 6.79 (0.27) | | 9.95 (0.08) | | 37 | | 31 | | 8.57 (0.27) | | 9.98 (0.23) | | 21 | 14 |
| Big trefoil | | Wild type | 7.34 (0.17) | | 9.38 (0.15) | | 32 | | 19 | | 10.04 (0.25) | | 10.72 (0.28) | | 7 | 6 |
| Lucerne | | Galaxy | 10.8 (0.15) | | 10.85 (0.081) | | 0 | | 1 | | 10.84 (0.12) | | 11.05 (0.11) | | 0 | 2 |
| Sainfoin | | CPI 63750 | 9.44 (0.17) | | 10.47 (0.18) | | 13 | | 10 | | 9.30 (0.09) | | 9.88 (0.09) | | 14 | 6 |
| Sainfoin | | Esky | 8.55 (0.18) | | 10.71 (0.15) | | 21 | | 20 | | 9.38 (0.14) | | 10.23 (0.11) | | 13 | 8 |
| Sainfoin | | Visnovsky | 8.79 (0.15) | | 10.23 (0.07) | | 19 | | 15 | | 8.67 (0.18) | | 10.17 (0.16) | | 20 | 15 |
| Plantain | | PLA60 | 8.67 (0.14) | | 8.93 (0.28) | | 20 | | 3 | | 9.93 (0.13) | | 9.66 (0.14) | | 8 | -6 |
| Plantain | | Svatojansky | 5.59 (0.16) | | 5.73 (0.13) | | 48 | | 3 | | 10.47 (0.09) | | 10.38 (0.05) | | 3 | -1 |
| Salad burnet | | PI 308861 | 7.93 (0.26) | | 9.92 (0.29) | | 27 | | 20 | | 7.05 (0.18) | | 8.98 (0.36) | | 35 | 22 |
| Salad burnet | | Sang 10 | 9.12 (0.24) | | 10.67 (0.17) | | 16 | | 15 | | 7.73 (0.41) | | 8.87 (0.36) | | 29 | 13 |
|  | **Factors** | | | **F-value** | | ***p*-value** | |  |  | **Factors** | | | | **F-value** | ***p*-value** |  |
|  | Cultivar | | | 423.5 | | <0.001 | |  |  | Cultivar x Harvest | | | | 94.8 | <0.001 |  |
|  | Treatment | | | 887.9 | | <0.001 | |  |  | Treatment x Harvest | | | | 33.0 | <0.001 |  |
|  | Harvest | | | 6.7 | | <0.1 | |  |  | Cultivar x Treatment x Harvest | | | | 5.4 | <0.001 |  |
|  | Cultivar x Treatment | | | 120.6 | | <0.001 | |  |  | |  | |  | |  |  |

**Supplementary Table S3**. Methane percentage in total gas (MP) from different species and cultivars across the two harvests with and without polyethylene glycol (PEG, a tannin binding agent) treatments and the reduction potential of the tested cultivars with respect to control. Red_MS refers to the reduction in MP from the cultivar when compared with lucerne, Red_PEG refers to reduction in MP from the cultivar in the absence of polyethylene glycol (-PEG) compared to the treatment with the addition of PEG (+PEG). Numbers between brackets correspond to the standard error of the mean (SEM).

| **Species** | | **Cultivar** | **Harvest 1** | | | | | | **Harvest 2** | | | | |
| --- | --- | --- | --- | --- | --- | --- | --- | --- | --- | --- | --- | --- | --- |
|  |  |  | **-PEG**  **(ml/200mg DM)** | **+PEG**  **(ml/200mg DM)** | **Red_MS (%)** | **Red_PEG (%)** | | **-PEG**  **(ml/200mg DM)** | | **+PEG**  **(ml/200mg DM)** | | **Red_MS (%)** | **Red_PEG (%)** |
| Chicory | | Plumato | 19.88 (0.37) | 19.64 (0.30) | 17 | -1 | | 20.52 (0.28) | | 20.32 (0.55) | | 15 | -1 |
| Chicory | | Spadona | 19.33 (0.08) | 19.49 (0.09) | 19 | 1 | | 20.72 (0.62) | | 20.87 (0.45) | | 14 | 1 |
| Sulla | | Grimaldi | 23.29 (0.38) | 22.76 (0.31) | 3 | -2 | | 23.64 (0.60) | | 23.17 (0.35) | | 2 | -2 |
| Sulla | | Sudda | 21.55 (0.35) | 21.57 (0.16) | 10 | 0 | | 22.90 (0.39) | | 22.73 (0.46) | | 5 | -1 |
| Birdsfoot trefoil | | Bull | 24.16 (0.24) | 23.47 (0.30) | -1 | -3 | | 23.47 (0.28) | | 22.08 (0.68) | | 3 | -6 |
| Birdsfoot trefoil | | Lotar | 24.58 (0.57) | 23.44 (0.24) | -2 | -4 | | 25.57 (0.33) | | 24.78 (0.59) | | -6 | -3 |
| Birdsfoot trefoil | | Rocco | 22.02 (0.16) | 21.92 (0.32) | 5 | 2 | | 25.62 (0.47) | | 24.95 (0.30) | | -6 | -3 |
| Big trefoil | | Lot 29 | 24.98 (0.81) | 23.26 (0.66) | -4 | -8 | | 24.03 (0.27) | | 22.88 (0.27) | | 0 | -5 |
| Big trefoil | | Wild type | 23.71 (0.86) | 22.44 (0.77) | 1 | -8 | | 23.66 (0.51) | | 23.28 (0.77) | | 2 | -2 |
| Lucerne | | Galaxy | 23.90 (0.36) | 22.59 (0.03) | 0 | -6 | | 24.2 (0.14) | | 23.35 (0.39) | | 0 | -3 |
| Sainfoin | | CPI 63750 | 22.13 (0.18) | 21.32 (0.22) | 7 | -4 | | 22.82 (0.42) | | 23.40 (0.25) | | 6 | 2 |
| Sainfoin | | Esky | 22.09 (0.25) | 21.82 (0.43) | 8 | -1 | | 23.73 (0.45) | | 23.93 (0.38) | | 2 | 1 |
| Sainfoin | | Visnovsky | 23.90 (0.44) | 22.03 (0.19) | 1 | -8 | | 23.10 (0.39) | | 22.80 (0.45) | | 4 | -1 |
| Plantain | | PLA60 | 19.07 (0.17) | 19.36 (0.31) | 20 | 1 | | 20.67 (0.29) | | 19.90 (0.33) | | 14 | -2 |
| Plantain | | Svatojansky | 17.19 (0.31) | 17.20 (0.52) | 28 | 0 | | 20.35 (0.13) | | 19.98 (0.15) | | 16 | -2 |
| Salad burnet | | PI 308861 | 20.70 (0.21) | 21.20 (0.38) | 13 | 2 | | 25.29 (0.53) | | 22.66 (0.88) | | -5 | -12 |
| Salad burnet | | Sang 10 | 20.61 (0.32) | 21.45 (0.33) | 14 | 4 | | 23.75 (0.42) | | 22.30 (0.30) | | 2 | -7 |
|  | **Factors** | | **F-value** | ***p*-value** |  |  | **Factors** | | | | **F-value** | ***p*-value** |  |
|  | Cultivar | | 505.22 | <0.0001 |  |  | Cultivar x Harvest | | | | 14.92 | <0.0001 |  |
|  | Treatment | | 13.78 | <0.05 |  |  | Treatment x Harvest | | | | 0.42 | 0.52 |  |
|  | Harvest | | 6.50 | <0.1 |  |  | Cultivar x Treatment x Harvest | | | | 3.15 | <0.0001 |  |
|  | Cultivar x Treatment | | 3.73 | <0.0001 |  |  | |  | |  | |  |  |
